# Transcription factor MAFB controls type I and II interferon response-mediated host immunity in *Mycobacterium tuberculosis*-infected macrophages

**DOI:** 10.3389/fmicb.2022.962306

**Published:** 2022-11-03

**Authors:** Haruka Hikichi, Shintaro Seto, Keiko Wakabayashi, Minako Hijikata, Naoto Keicho

**Affiliations:** ^1^Department of Pathophysiology and Host Defense, The Research Institute of Tuberculosis, Japan Anti-Tuberculosis Association, Tokyo, Japan; ^2^Department of Basic Mycobacteriosis, Nagasaki University Graduate School of Biomedical Sciences, Nagasaki, Japan; ^3^Vice Director, The Research Institute of Tuberculosis, Japan Anti-Tuberculosis Association, Tokyo, Japan

**Keywords:** Mycobacterium tuberculosis, macrophage, MAFB, interferon response, RNA sequencing, gene set enrichment analysis

## Abstract

*MAFB*, v-maf avian musculoaponeurotic fibrosarcoma oncogene homolog B, has been identified as a candidate gene for early tuberculosis (TB) onset in Thai and Japanese populations. Here, we investigated the genome-wide transcriptional profiles of MAFB-knockdown (KD) macrophages infected with *Mycobacterium tuberculosis* (*Mtb*) to highlight the potential role of MAFB in host immunity against TB. Gene expression analysis revealed impaired type I and type II interferon (IFN) responses and enhanced oxidative phosphorylation in MAFB-KD macrophages infected with *Mtb*. The expression of inflammatory chemokines, including IFN-γ-inducible genes, was confirmed to be significantly reduced by knockdown of MAFB during *Mtb* infection. A similar effect of MAFB knockdown on type I and type II IFN responses and oxidative phosphorylation was also observed when *Mtb*-infected macrophages were activated by IFN-γ. Taken together, our results demonstrate that MAFB is involved in the immune response and metabolism in *Mtb*-infected macrophages, providing new insight into MAFB as a candidate gene to guide further study to control TB.

## Introduction

Tuberculosis (TB) was the leading cause of death from a single infectious agent, *Mycobacterium tuberculosis* (*Mtb*), until coronavirus disease 2019 (COVID-19) emerged. Reduced access to TB services, including early diagnosis and treatment, has resulted in an increased number of TB deaths (Dheda et al., 2022), an estimated 1.5 million in 2020 (WHO, 2021). To further accelerate the progression of global TB control toward the end of the TB epidemic, the development of new TB diagnostics, drugs and vaccines, along with basic TB research, is required.

Host genetic factors are important for susceptibility to TB, together with environmental and bacterial factors (Abel et al., 2018). Genome-wide association studies (GWASs) allow unbiased identification of numerous genetic variants associated with TB susceptibility and provide insights into the molecular mechanisms of host immunity against TB (Dallmann-Sauer et al., 2018). Among more than 10 GWASs reporting TB-associated loci to date, Mahasirimongkol et al. demonstrated that v-maf avian musculoaponeurotic fibrosarcoma oncogene homolog B (*MAFB*) was located in the neighborhood of a single nucleotide polymorphism associated with TB onset younger than 45 years old in Thai and Japanese populations (Mahasirimongkol et al., 2012). Further study demonstrated that *MAFB* was differentially expressed in active TB patients compared with healthy controls (Satproedprai et al., 2015), suggesting that MAFB has a role in TB pathogenesis.

MAFB is a transcription factor and a member of the large Maf family that is selectively expressed by monocytes and macrophages to induce monocyte–macrophage differentiation (Kataoka et al., 1994; Kelly et al., 2000). This gene is involved in differentiation or development in various cells, tissues and organs, and its function is related to macrophages (Hamada et al., 2020). Regarding infection, MAFB has been associated with infectious diseases, including chronic hepatitis C, Zika virus infection and leprosy (Orlova et al., 2013; Moni and Lio, 2017; Liu et al., 2019). MAFB has also been reported to be involved in type I interferon (IFN) regulation, which drives host vulnerability to viral infection (Kim and Seed, 2010).

In this study, we investigated the involvement of MAFB in gene regulation in *Mtb*-infected macrophages. We knocked down MAFB in *Mtb*-infected macrophages and surveyed genome-wide transcriptional profiles by messenger RNA sequencing (mRNA-seq). In *Mtb*-infected macrophages, knockdown of MAFB impaired the expression of genes related to type I and II IFN responses along with the expression of inflammatory chemokine genes, including IFN-γ-inducible genes, while knockdown induced the expression of genes related to oxidative phosphorylation. These trends were also observed when macrophages were activated by IFN-γ. Taken together, we suggest that MAFB is associated with the immune response and metabolism of *Mtb*-infected macrophages.

## Materials and methods

### Cells

Human THP-1 cells were obtained from RIKEN BRC and cultured in RPMI-1640 medium (Sigma–Aldrich) supplemented with 10% fetal bovine serum (Nichirei Bioscience), 100 U/ml penicillin and 100 μg/ml streptomycin (complete medium). For activation, THP-1 cells (2 × 10^5^ cells/ml) were seeded in complete medium containing 10 ng/ml phorbol myristate acetate (PMA; Sigma) in six-well collagen-coated tissue culture plates (AGC Techno Glass) and incubated for 24 h. Adherent THP-1 cells were washed twice with complete medium and then incubated in complete medium without antibiotics for an additional 48 h. For stimulation with IFN-γ, macrophages were subsequently treated with IFN-γ at 10 ng/ml (Biolegend).

### RNA interference

Small interfering RNAs (siRNAs) targeting *MAFB* gene were synthesized by Sigma–Aldrich. The siRNA sequences were as follows: mafb1, sense 5´-CUGUCUGUCAGAGUUCGGATT-3′, antisense 5´-UCCGAACUCUGACAGACAGTT-3′; mafb2, sense 5´-CUGCUUUGCUGCCCGGAGATT-3′, antisense 5′- UCUCCGGGCAGCAAAGCAGTT-3′; Mission siRNA Universal Negative Control (Sigma–Aldrich) was used as the control. Transfection of macrophages with siRNA duplexes was performed using Lipofectamine RNAiMAX (Invitrogen) according to the manufacturer’s instructions. Lipid-RNA complexes were added at the wash step of PMA activation. After transfection for 48 h, the medium was replaced with complete medium without antibiotics, prior to subsequent experiments.

### Quantitative real-time reverse transcriptase-polymerase chain reaction

Total RNA was extracted using an RNeasy Mini Kit (Qiagen) and reverse transcribed into cDNA using Prime Script Reverse Transcriptase (Takara). qRT–PCR was performed by using TaqMan Universal Master Mix (Thermo Fisher). The primers and probes used in this study are listed in Supplementary Table S1. The minus threshold cycle (Ct) value of target genes normalized to that of *GAPDH* was calculated and compared to that of the control group.

### Immunoblot analysis and ELISA

MAFB-KD or control macrophages were washed with ice-cold PBS and lysed with lysis buffer containing 50 mM HEPES (pH 7.2), 150 mM NaCl, 1% Triton X-100 and Roche Complete EDTA Free Protease Inhibitor Cocktail. Cell lysates were separated by sodium dodecyl sulfate–polyacrylamide gel electrophoresis (SDS–PAGE) and then subjected to immunoblot analysis using an anti-MAFB (1:1000 v/v; Proteintech, #20189-1-AP) or anti-GAPDH (1:6000 v/v; Medical and Biological Laboratories, #M171-3) antibody. Band intensities were quantified using Image Lab Software version 6.0 (Bio-Rad).

The concentrations of secreted MCP-1 and IP-10 from MAFB-KD and control macrophages infected with *Mtb* were measured by a Human CCL2/MCP-1 Quantikine ELISA Kit and a Human CXCL10/IP-10 Quantikine ELISA Kit (R&D Systems), respectively. The culture media from *Mtb*-infected macrophages were collected at 4 and 24 h postinfection (p.i.), followed by filtration with a 0.45-μm pore size filter (Toyo Roshi Kaisha).

### *Mtb* infection

*Mtb* strain Erdman was grown to midlogarithmic phase in 7H9 medium supplemented with 10% Middlebrook ADC (BD Biosciences), 0.5% glycerol and 0.05% Tween 80 (Mycobacterium medium) at 37°C (Seto et al., 2012). DsRed-expressing *Mtb* was grown in Mycobacterium medium containing kanamycin at 50 μg/ml as described previously (Seto et al., 2012). For preparation of the single cell suspension, growing bacterial cultures were filtered through a 5-μm pore size filter (Pall Corporation) as described previously (Yamada et al., 2001; Furuuchi et al., 2022). Aliquots of the filtrated bacterial solutions were stored at-80°C until use. The number of bacteria was determined by a colony-forming-unit (CFU) assay using plates with 7H10 agar base medium supplemented with 10% Middlebrook OADC (BD Biosciences) and 0.5% glycerol. PMA-treated THP-1 cells were infected with *Mtb* at a multiplicity of infection of 1 in complete medium without antibiotics for 24 h and simultaneously stimulated with 10 ng/ml IFN-γ.

### Phagocytosis and CFU analyses

For phagocytosis analysis, *Mtb*-infected MAFB-KD and control macrophages grown on coverslips in 12-well plates were fixed with 3% paraformaldehyde in PBS at 4°C for 24 h, washed with PBS three times, and mounted on microscope slides using Vectashield Antifade Mounting Medium with DAPI (Vector Laboratories). Florescence microscopy was performed with an Olympus IX81 microscope equipped with a DP74 camera (Olympus). The phagocytic index was determined as a ratio of *Mtb*-infected macrophages to all macrophages per field and expressed. Fields with fewer than five macrophages were removed.

For CFU analysis, the culture medium was removed from *Mtb*-infected macrophages at 24 or 48 h p.i., and the cells were collected and lysed with 1% SDS in PBS. Samples were serially diluted with Mycobacterium complete medium (7H9) and inoculated in duplicate onto plates with 7H10 agar base medium supplemented with 10% Middlebrook OADC and 0.5% glycerol. CFU values were calculated as the means of the two plates at each dilution.

### mRNA-seq

The quality and quantity of isolated RNA from macrophages were assessed using a Nanodrop 1,000 Spectrophotometer or Qubit 3.0 Fluorometer (Thermo Fisher Scientific) and an RNA 6000 Nano Kit on an Agilent 2,100 Bioanalyzer (Agilent Technologies). Two micrograms of total RNA with an RNA integrity number (RIN) greater than 7 was used to construct cDNA libraries using a TruSeq Stranded mRNA library kit (Illumina). All cDNA libraries were checked for quality using a DNA 1000 Kit on an Agilent 2,100 Bioanalyzer and quantified with a Qubit 3.0 Fluorometer and a GenNext NGS Library Quantification Kit (Toyobo). The libraries were sequenced on an Illumina NextSeq 500 to generate more than 25 million 75 base-long paired-end reads per library. Raw sequence data have been deposited in the DRA database under the accession number DRA014120.

### Data processing

RNA-seq data were processed as described previously (Seto et al., 2022). Briefly, raw reads were processed with Trim Galore v0.6.6 for read-quality trimming.^1^ The processed reads were then aligned with HISAT2 v2.2.1 (Kim et al., 2019) against the human genome hg38. Gene abundance estimation was performed with featureCounts v2.0.1 (Liao et al., 2014). More than 25 million high-quality clean reads were mapped to the human reference genome, and the mapping rate of each sample was greater than 98% as determined by the Picard toolkit.^2^

Differential gene expression analysis was performed with edgeR v3.32.1 (Robinson et al., 2010) using generalized linear models and quasi-likelihood tests (Chen et al., 2016). Differentially expressed genes (DEGs) were identified using the cutoff *p* values with a false discovery rate (FDR) of 0.01 provided by edgeR (Supplementary Table S2). The DEGs were further utilized for Gene Ontology (GO) enrichment analysis using clusterProfiler v3.18.1 to visualize enriched biological process (BP) terms (GOBP; Yu et al., 2012).

To assess whether a genetically defined genome was significantly different between the two phenotypes, gene set enrichment analysis (GSEA; Subramanian et al., 2005) was performed with the fgsea package v1.16.0 (Korotkevich et al., 2021) with hallmark gene sets obtained from the Molecular Signatures Database (MSigDB). An adjusted *p* value of less than 0.25 was considered significant. Z-score was calculated to test the clustering among selected DEGs, and the data were visualized with the pheatmap package v1.0.12.^3^

## Results

### MAFB expression in THP-1 cells

**Figure 1:**
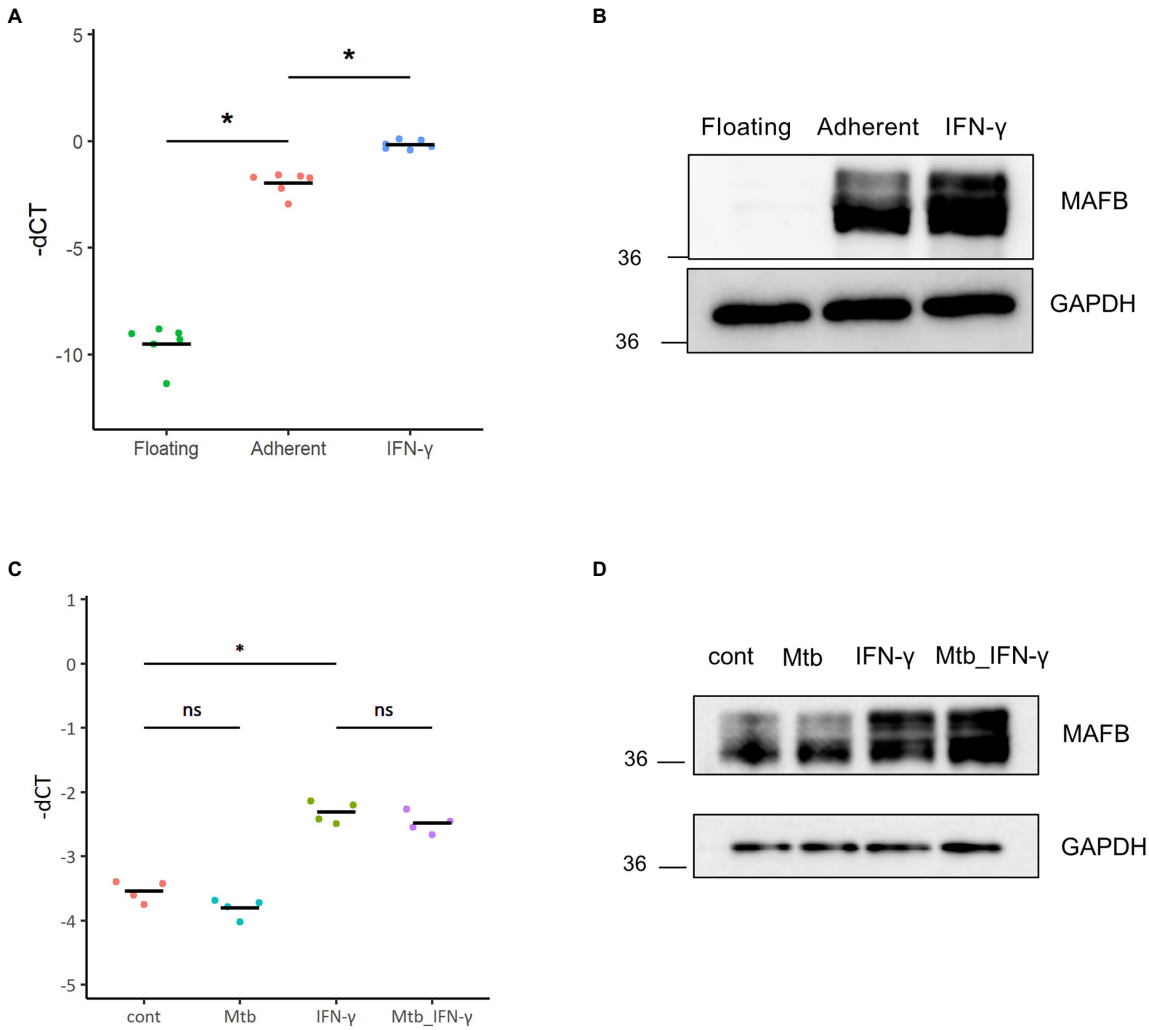
MAFB expression in THP-1 cells. **(A)** The mRNA expression level of MAFB was examined by qRT–PCR in untreated (floating), PMA-treated (adherent), or PMA- and IFN-γ-treated (IFN-γ) THP-1 cells. The minus CT values of *MAFB* normalized to *GAPDH* (-dCT) are shown. * *p* < 10^−4^ using ANOVA with Tukey’s multiple comparison test. **(B)** Immunoblot analysis of MAFB and GAPDH. MAFB was detected in floating, adherent, and IFN-γ THP-1 cells. Whole-cell lysates were subjected to SDS–PAGE, followed by immunoblot analysis using the indicated antibodies. **(C)** The mRNA expression level of *MAFB* in control (cont), IFN-γ-treated (IFN-γ), *Mtb*-infected (Mtb), or *Mtb*-infected and IFN-γ-treated (Mtb_IFN-γ) THP-1 macrophages. * *p* < 10^−4^, ns, not significant using ANOVA with Tukey’s multiple comparison test. **(D)** Immunoblot analysis of MAFB and GAPDH in control (cont), IFN-γ-treated (IFN-γ), *Mtb*-infected (Mtb), or *Mtb*-infected and IFN-γ-treated (Mtb_IFN-γ) THP-1 macrophages.

**Figure 2:**
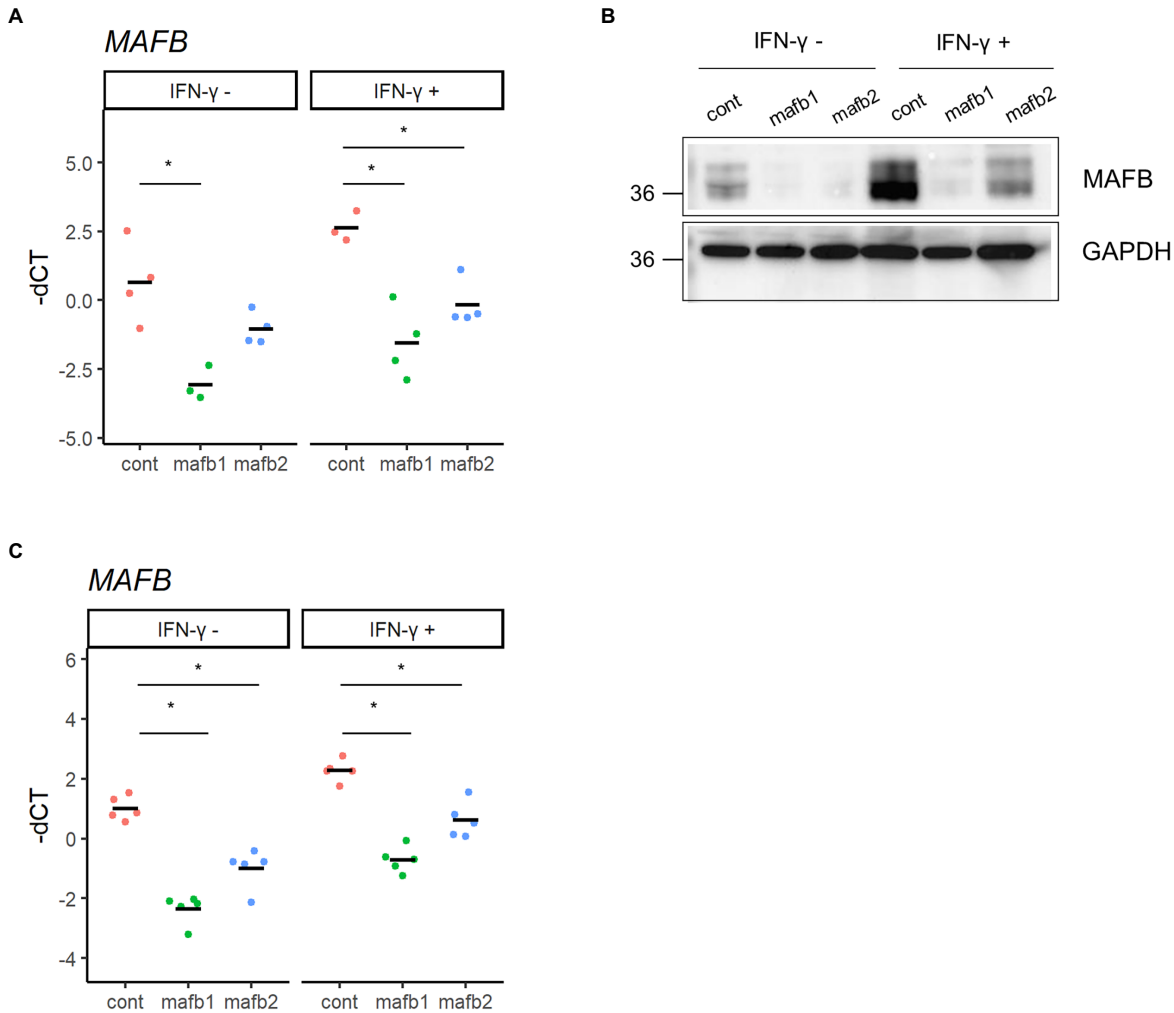
Knockdown of MAFB in THP-1 macrophages. **(A,B)** MAFB knockdown by siRNA. PMA-treated THP-1 cells were transfected with a MAFB-targeting (mafb1, mafb2) or nontargeting control siRNA (cont). The expression levels of *MAFB* mRNA and protein were examined by qRT–PCR **(A)** and immunoblot analysis **(B)**, respectively. These expression levels were also examined in IFN-γ-stimulated macrophages. For qRT–PCR data, the-dCT values are shown. *p < 0.05 using ANOVA with Dunnett’s test. **(C)** MAFB expression upon *Mtb* infection. PMA-treated THP-1 cells transfected with mafb1, mafb2 or control siRNAs were infected with *Mtb* for 24 h. The *MAFB* mRNA level was examined by qRT–PCR. Gene expression was also examined in IFN-γ-stimulated cells. The-dCT values are shown. *p <  0.05 using one-way ANOVA with Dunnett’s test.

THP-1 cells, a human monocytic leukemia cell line, are widely accepted as an *in vitro* infection model to study the immune response against intracellular bacteria, including *Mtb* (Chanput et al., 2014). In this study, we employed THP-1 cells to assess the function of MAFB in monocyte/macrophage lineage cells infected with *Mtb*. We examined the expression of MAFB in THP-1 cells by qRT–PCR and immunoblot analyses. In unstimulated cells, MAFB was detected at low levels. MAFB was greatly induced when cells were differentiated into macrophages by treatment with PMA or stimulation with IFN-γ after PMA treatment (Figures 1A,B). In PMA-stimulated THP-1 cells, the expression level of MAFB was not changed after *Mtb* infection, even under IFN-γ stimulation (Figures 1C,D). To decrease gene expression, we designed two sets of siRNAs targeting MAFB and transfected THP-1-derived macrophages with the siRNA duplexes. We verified the knockdown efficiency by qRT–PCR and immunoblot analyses and confirmed that MAFB mRNA and protein expression was markedly decreased (approximately 70–90%) by the MAFB-targeting siRNA duplexes (Figures 2A,B). We also examined the knockdown efficiency in *Mtb*-infected macrophages and confirmed that both siRNAs significantly silenced MAFB expression in both the presence and absence of IFN-γ (Figure 2C). These results confirmed the stability of MAFB knockdown in our experimental model.

**Figure 3:**
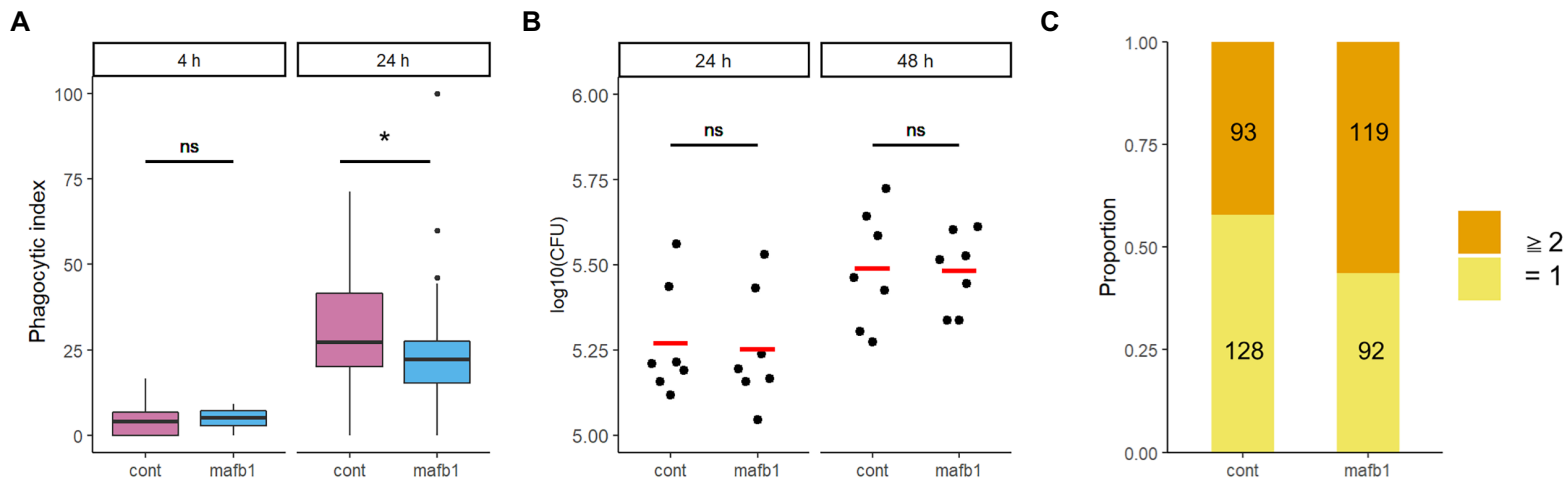
Phagocytosis and intracellular proliferation of *Mtb* within THP-1 macrophages. PMA-treated THP-1 cells transfected with mafb1 (mafb1) or control siRNA (cont) were infected with *Mtb*. **(A)** The phagocytic index represents the frequency of *Mtb*-infected macrophages per field observed by florescence microscopy at 4 h and 24 h p.i. * *p* = 0.01, ns, not significant using Wilcoxon rank sum test. **(B)** Colony-forming unit (CFU) values were determined at 24 and 48 h p.i. The dots indicate the log 10-fold CFU values from seven independent experiments. The mean CFU for each condition is shown as a red bar. ns, not significant using Student’s *t* test. **(C)** Number of the intracellular bacilli within phagocytosed macrophages. The number of DsRed-expressing *Mtb* bacilli within macrophages at 24 h p.i. was counted. The numbers of macrophages containing one bacillus (yellow) and two or more bacilli (orange) are indicated.

We evaluated the effect of MAFB depletion on phagocytosis and survival of intracellular bacilli in THP-1 macrophages. The phagocytosis ability of *Mtb* was not changed between MAFB-KD and control macrophages at 4 h p.i. (*p* = 0.34), while it was impaired in MAFB-KD macrophages at 24 h p.i. (*p* = 0.01; Figure 3A), suggesting that MAFB regulates the phagocytosis of *Mtb* into macrophages. The CFU assay demonstrated that the survival of intracellular bacilli exhibited no significant difference between MAFB-KD and control macrophages (*p* = 0.877 at 24 h p.i., *p* = 0.777 at 48 h p.i.; Figure 3B), implying that *Mtb* proliferation was enhanced within MAFB-KD macrophages even though phagocytosis was impaired. We counted bacterial numbers within MAFB-KD or control macrophages and found that the proportion of MAFB-KD macrophages containing two or more bacilli among *Mtb*-containing macrophages was greater than that of control macrophages (*p* = 0.004, Fisher’s exact test; Figure 3C). Taken together, these results suggest that MAFB regulates the various intracellular pathways involved in the response to *Mtb* infection in human macrophages.

### Genome-wide transcriptional profiling in THP-1 macrophages infected with *Mtb*

**Figure 4:**
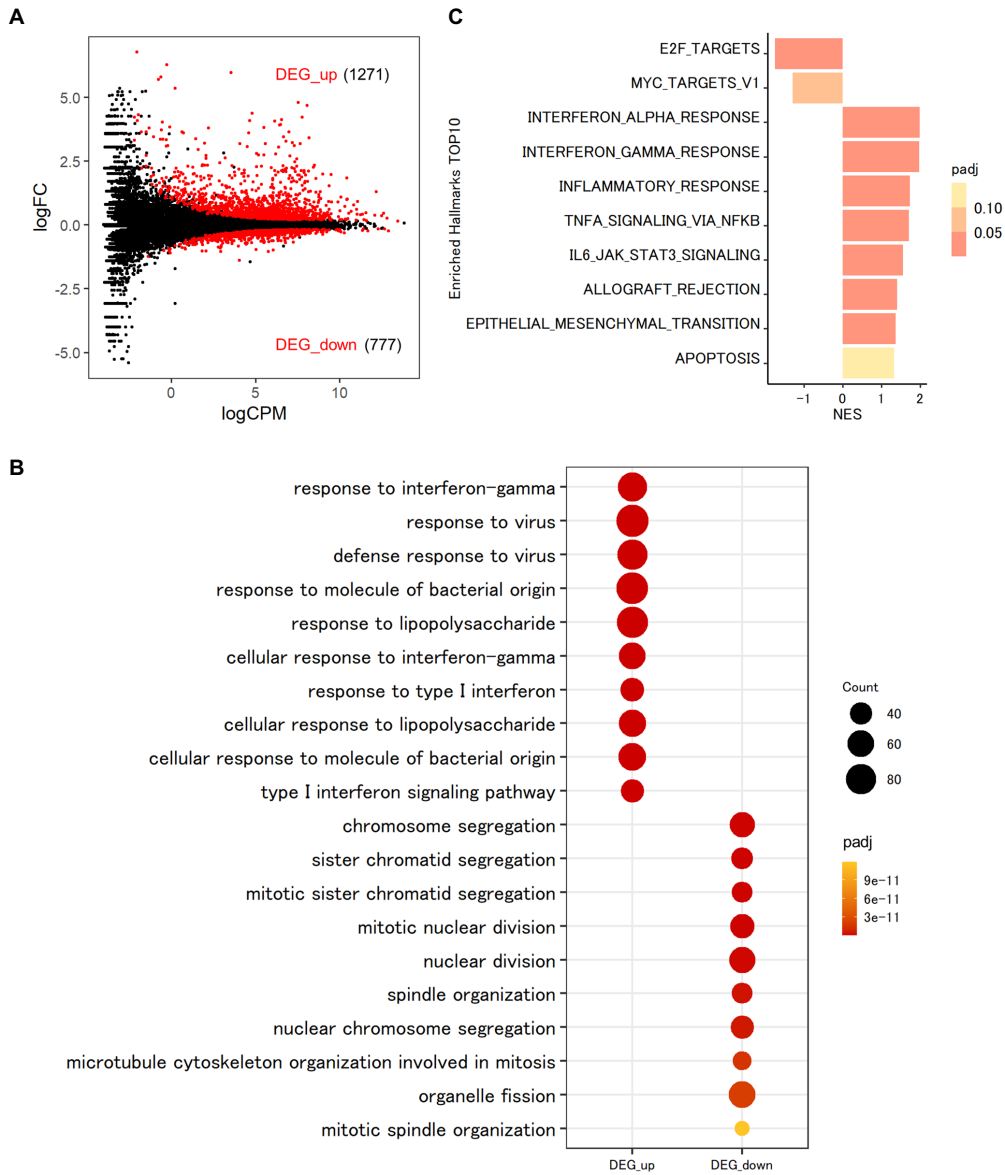
Transcriptomic analysis of THP-1 macrophages infected with *Mtb.* **(A)** Impact of *Mtb* infection on global gene expression in macrophages. PMA-treated THP-1 cells transfected with control siRNA were infected with *Mtb* for 24 h. RNA was extracted and subjected to messenger RNA sequencing (mRNA-seq). MA plot showing the upregulated differentially expressed genes (DEGs, DEG_up) and downregulated DEGs (DEG_down) of *Mtb*-infected macrophages compared with uninfected control macrophages marked in red (FDR < 0.01). **(B)** Enrichment analyses of Gene Ontology related to biological process (GOBP) terms for *Mtb*-infected macrophages. The 10 most significant GOBP terms for DEG_up and DEG_down are shown. Count, gene count. Padj, adjusted *p* value. **(C)** Gene set enrichment analysis (GSEA) of *Mtb*-infected macrophages. The 10 most significantly enriched hallmarks are shown. The color scale indicates the adjusted *p* value, and the bar size in the histogram indicates the normalized enrichment score (NES). FC, fold change. CPM, counts per million. NES, normalized enrichment score. Padj, adjusted *p* value.

To assess the function of MAFB in regulating the intracellular pathways in *Mtb*-infected THP-1 macrophages, we conducted transcriptomic analysis. To explore the transcriptomic features of macrophages after *Mtb* infection, we first compared the gene expression profiles between *Mtb*-infected macrophages and noninfected control macrophages. In total, 2048 DEGs were identified in *Mtb*-infected macrophages, among which 1,271 were upregulated and 777 were downregulated (FDR < 0.01; Figure 4A; Supplementary Table S2). GO analysis is widely used to capture biological information from large quantities of data generated by genome-wide expression studies, including mRNA-seq. By grouping genes based on the annotated GO terms, GO analysis provides us with predominant biological themes in a collection of genes (Ashburner et al., 2000). GOBP enrichment analysis of the DEGs showed that 1,127 GO terms related to the immune response, including response to interferon gamma (GO:0034341), response to virus (GO:0009615), defense response to virus (GO:0051607) and response to molecule of bacterial origin (GO:0002237), were enriched in upregulated DEGs, as previously reported (Wu et al., 2012; Pu et al., 2021; Figure 4B). Moreover, 355 GO terms, such as chromosome segregation (GO:0007059), sister chromatid segregation (GO:0000819), mitotic sister chromatid segregation (GO:0000070), mitotic nuclear division (GO:0140014), and nuclear division (GO:0000280), were enriched in downregulated DEGs (Figure 4B). To identify the general features of all expressed genes, we performed GSEA using the GSEA hallmark pathway database. As shown in Figure 4C, genes related to type I and type II IFN responses and tumor necrosis factor (TNF) signaling pathways were upregulated, on the other hand, those related to cell proliferation were downregulated in *Mtb*-infected macrophages compared with uninfected control macrophages. These results agreed with those of GOBP enrichment analysis of the DEGs.

### Genome-wide transcriptional profiling in MAFB-KD macrophages infected with *Mtb*

**Figure 5:**
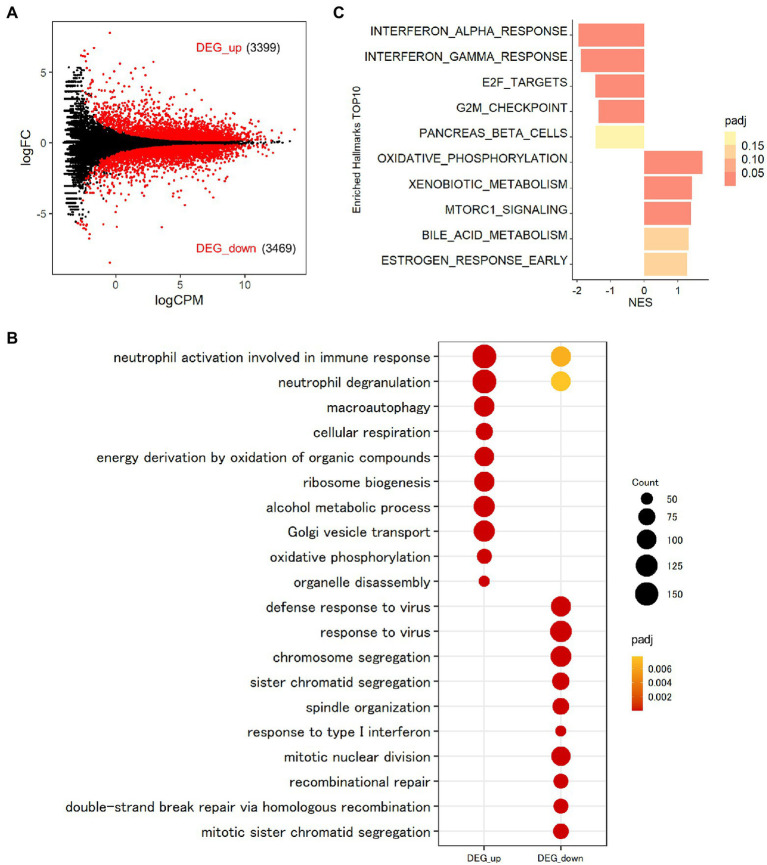
Transcriptomic analysis of MAFB-KD macrophages infected with *Mtb.* **(A)** Impact of MAFB depletion during *Mtb* infection on global gene expression in macrophages. PMA-treated THP-1 cells transfected with mafb1, or control siRNA were infected with *Mtb* for 24 h. RNA was extracted and subjected to mRNA-seq. MA plot showing the upregulated DEGs (DEG_up) and downregulated DEGs (DEG_down) in *Mtb*-infected MAFB-KD compared with control macrophages marked in red (FDR < 0.01). **(B)** GOBP enrichment analyses of DEG_up and DEG_down in *Mtb*-infected MAFB-KD macrophages. The 10 most significant GOBP terms are shown. Count, gene count. Padj, adjusted *p* value. **(C)** GSEA of *Mtb*-infected MAFB-KD macrophages. Enriched hallmarks are shown (FDR < 0.25). The color scale indicates the adjusted *p* value, and the bar size in the histogram indicates the normalized enrichment score (NES). FC, fold change. CPM, counts per million. NES, normalized enrichment score. Padj, adjusted *p* value.

**Figure 6:**
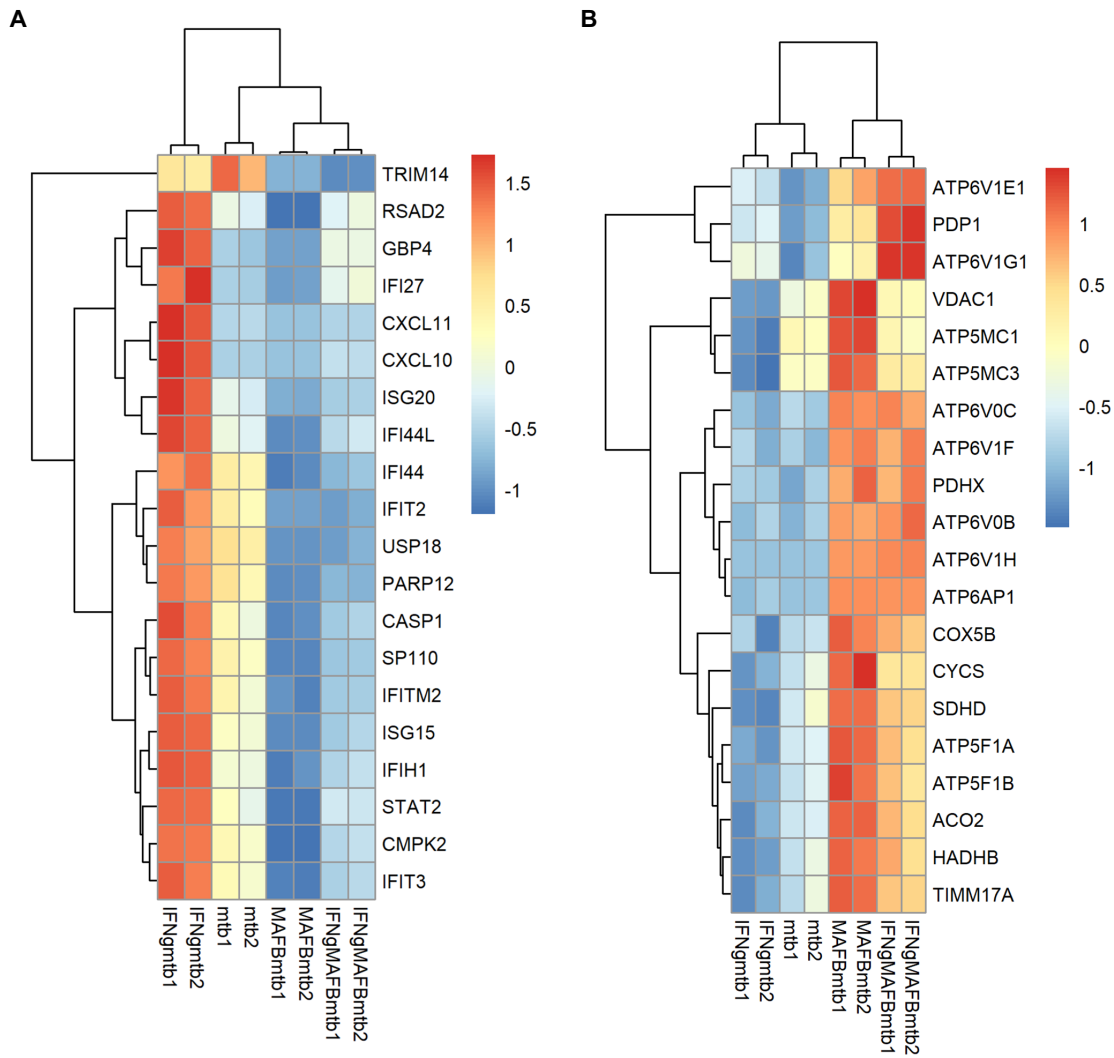
Heatmap visualization of the expression of the key leading edge genes. The leading edge genes were obtained from the enriched GSEA hallmark gene sets. From these genes, the 20 genes with the highest FDRs were selected and subjected to hierarchical clustering to compare gene expression between the conditions. The heatmap shows the z score-ranked mRNA expression patterns of leading edge genes of the hallmark IFN-alpha and IFN-gamma response **(A)** or the hallmark oxidative phosphorylation **(B)**.

To investigate the roles of MAFB in the context of *Mtb* infection, we compared the gene expression profiles of MAFB-KD macrophages to those of control macrophages upon *Mtb* infection. In *Mtb*-infected MAFB-KD macrophages transfected with MAFB siRNA mafb1, 6,768 DEGs (FDR < 0.01) were identified relative to *Mtb*-infected control macrophages (Figure 5A; Supplementary Table S2). GSEA revealed that genes related to type I and type II IFN responses, E2F targets and the G2/M checkpoint were downregulated, but those related to oxidative phosphorylation, xenobiotic metabolism, and mTORC1 signaling were upregulated in MAFB-KD macrophages compared to control macrophages (Figure 5C). GOBP enrichment analysis showed results corresponding to those of GSEA; Response to virus (GO:0009615), defense response to virus (GO:0051607), and response to type 1 interferon (GO:0034340) were enriched in downregulated DEGs. Oxidative phosphorylation (GO:0006119) and cellular respiration (GO:0045333) were enriched in upregulated DEGs (Figure 5B). We confirmed that transfection of control siRNA did not affect the expression of type I or type II IFN-related genes (data not shown). To further explore the role of MAFB when macrophages are activated by IFN-γ, we examined the gene expression profiles of *Mtb*-infected macrophages stimulated with IFN-γ and compared them to those of *Mtb*-infected control macrophages stimulated with IFN-γ. A total of 6,374 DEGs were identified in this comparison (Supplementary Figure S1; Supplementary Table S2). Similar trends were observed in GOBP enrichment analysis and GSEA in *Mtb*-infected MAFB-KD macrophages stimulated with IFN-γ: the genes related to type I and II IFN responses were downregulated, and those related to oxidative phosphorylation were upregulated. To further analyze the impact of MAFB knockdown on genes related to type I and type II IFN signaling pathways and oxidative phosphorylation, the 20 most significant genes were extracted for hierarchical clustering. For the hallmark IFN-gamma and the hallmark IFN-alpha, significant genes shared by the gene sets of both hallmarks were obtained. Of the genes, 19 were clustered, showing strong upregulation in *Mtb*-infected IFN-γ-activated macrophages and downregulation in *Mtb*-infected MAFB-KD macrophages. IFN-γ activation did not reverse the effect of MAFB knockdown (Figure 6A). For oxidative phosphorylation, genes were induced by MAFB knockdown independent of IFN-γ stimulation (Figure 6B).

As MAFB is a transcription factor that binds to Maf recognition elements (MAREs), we investigated whether DEGs in MAFB-KD macrophages infected with *Mtb* in our study are direct target genes bound by MAFB. Compared with the data from chromatin immunoprecipitation sequencing (ChIP-seq) of MafB for mouse macrophages (Dieterich et al., 2015), of 6,768 DEGs, 2,726 genes (40.3%) were considered direct targets of MAFB (Supplementary Figure S2A), including genes related to IFN responses and metabolism. GOBP analysis showed that these direct target genes for MAFB in DEGs are enriched cellular responses, such as actin filament organization, regulation of protein-containing complex assembly, and response to molecules of bacterial origin (Supplementary Figure S2B), suggesting that these processes including the sensing and phagocytosis of *Mtb* are the most upstream events mediated by MAFB in the course of *Mtb* infection.

### Validation of mRNA-seq results by qRT–PCR

**Figure 7:**
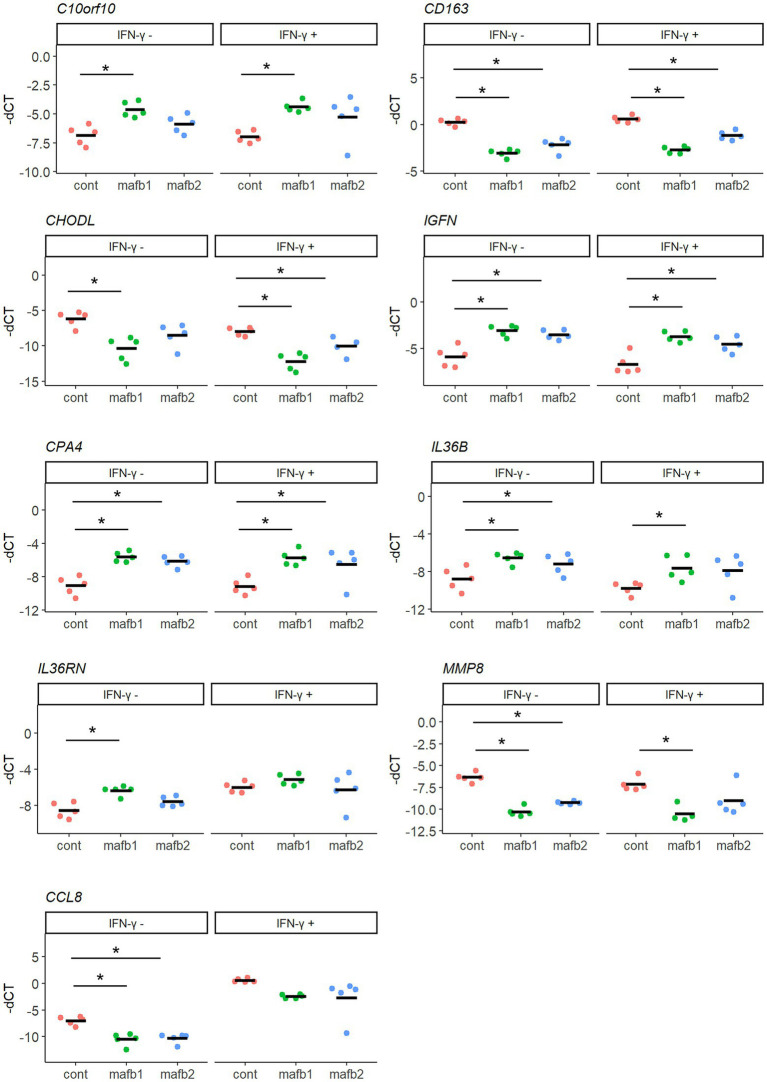
qRT–PCR validation of DEGs characterized by mRNA-seq. Nine genes with the lowest FDRs were selected from DEG_up and DEG_down in *Mtb*-infected MAFB-KD macrophages and the mRNA expression was validated by qRT–PCR. PMA-treated THP-1 cells were transfected with mafb1, mafb2 siRNAs or control siRNA, followed by *Mtb* infection for 24 h in the presence and absence of IFN- γ. RNA was extracted and subjected to qRT–PCR. The-dCT values of the indicated genes are shown. The data represent the results from five biological replicates. ns, not significant. *p < 0.05 using one-way ANOVA with Dunnett’s test.

To validate the mRNA-seq results, we selected nine of the most significant genes that are generally known for their function in macrophages and evaluated their mRNA expression by qRT–PCR. As upregulated genes upon MAFB knockdown, *C10orf10*, *CPA4*, *IL36RN*, *IGFN*, and *IL36B* were examined. As downregulated genes upon MAFB knockdown, *CHODL*, *CCL8*, *CD163*, and *MMP8* were examined. The expression of these key genes was significantly induced or reduced in both IFN-γ-stimulated MAFB-KD macrophages and unstimulated MAFB-KD macrophages (Figure 7). These results support the mRNA-seq results.

### MAFB knockdown downregulates IFN-γ-inducible inflammatory genes

**Figure 8:**
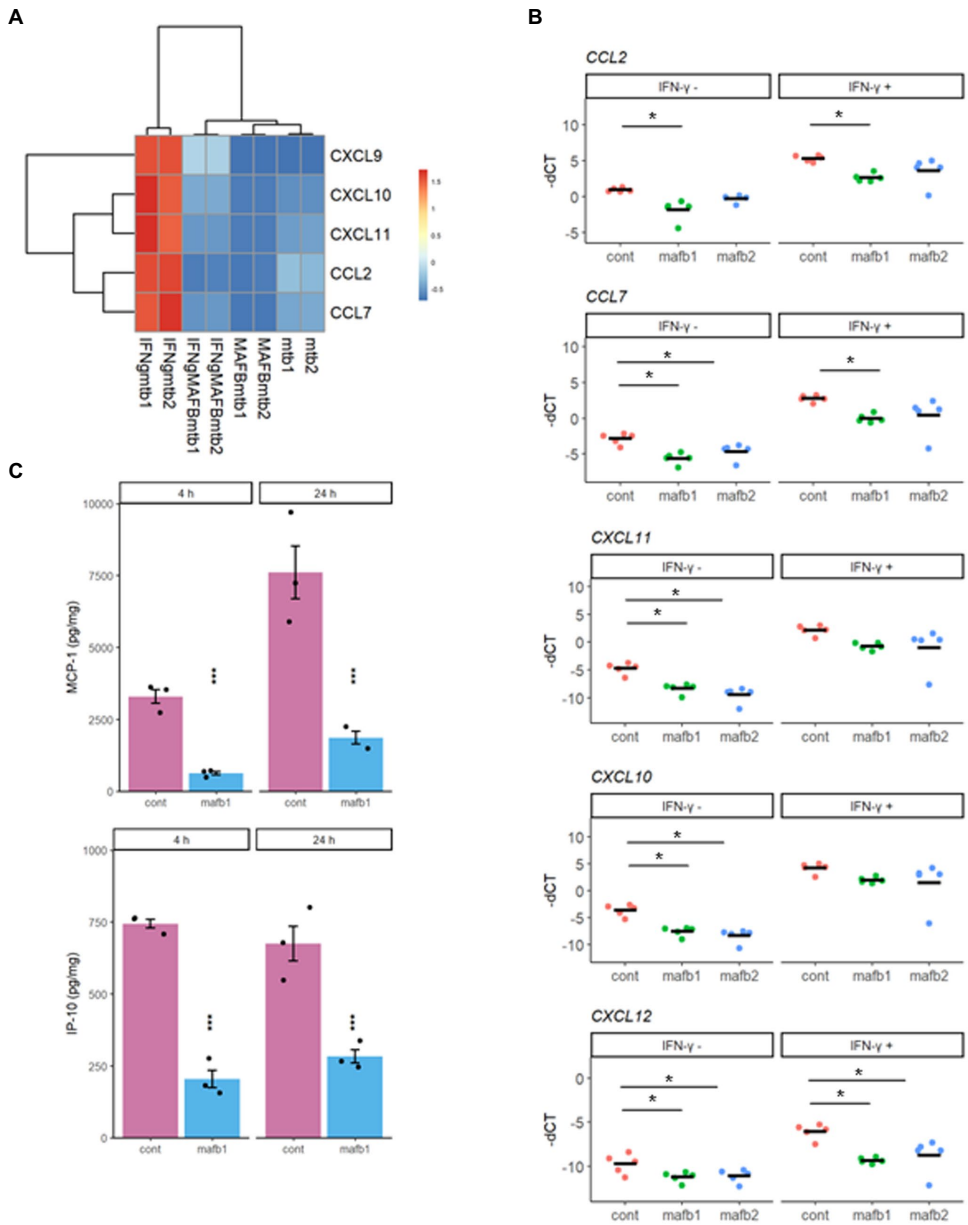
Chemokine activity in MAFB-KD macrophages. **(A)** Heatmap showing the z score-ranked mRNA expression patterns of five inflammatory chemokines. **(B)** qRT–PCR validation of inflammatory chemokines. MAFB-KD (mafb1, mafb2) and control (cont) macrophages were infected with *Mtb* for 24 h in the presence or absence of IFN-γ. RNA was extracted for qRT–PCR. The-dCT values are shown. *p < 0.05 using one-way ANOVA with Dunnett’s test. ns, not significant. **(C)** ELISA for secreted chemokines in *Mtb*-infected MAFB-KD macrophages. MAFB-KD (mafb1) and control (cont) macrophages were infected with *Mtb* for 4 and 24 h. MCP-1 and IP-10 levels were measured by ELISA. * *p* < 0.05 using Student’s *t* test.

To analyze the detailed effect of MAFB knockdown on IFN-γ activation, we examined the activities of IFN-γ-inducible chemokines. Hierarchical clustering of the differentially expressed IFN-γ-inducible chemokines (*CXCL9*, *CXCL10*, *CXCL11*, *CCL2*, and *CCL7*) showed that their gene expression was highly upregulated in *Mtb*-infected IFN-γ-activated macrophages and downregulated in MAFB-KD macrophages (Figure 8A). To verify the mRNA-seq results, we quantified the mRNA expression of these genes by qRT–PCR. All of these genes were significantly downregulated in MAFB-KD macrophages infected with *Mtb* compared to control macrophages. Furthermore, *CCL2*, *CCL7*, and *CXCL12* were also significantly downregulated upon IFN-γ stimulation (Figure 8B). We also verified the mRNA-seq results by ELISA for CCL2 and CXCL10. The secretion levels of both MCP-1 (CCL2) and IP-10 (CXCL10) were significantly reduced in MAFB-KD macrophages infected with *Mtb* (Figure 8C), confirming that reduced IFN-γ related gene expression by MAFB knockdown consequently leads to decreased chemokine secretion.

### Response to *Mtb* infection in MAFB-KD macrophages at the different time points for *MAFB* gene depletion

**Figure 9:**
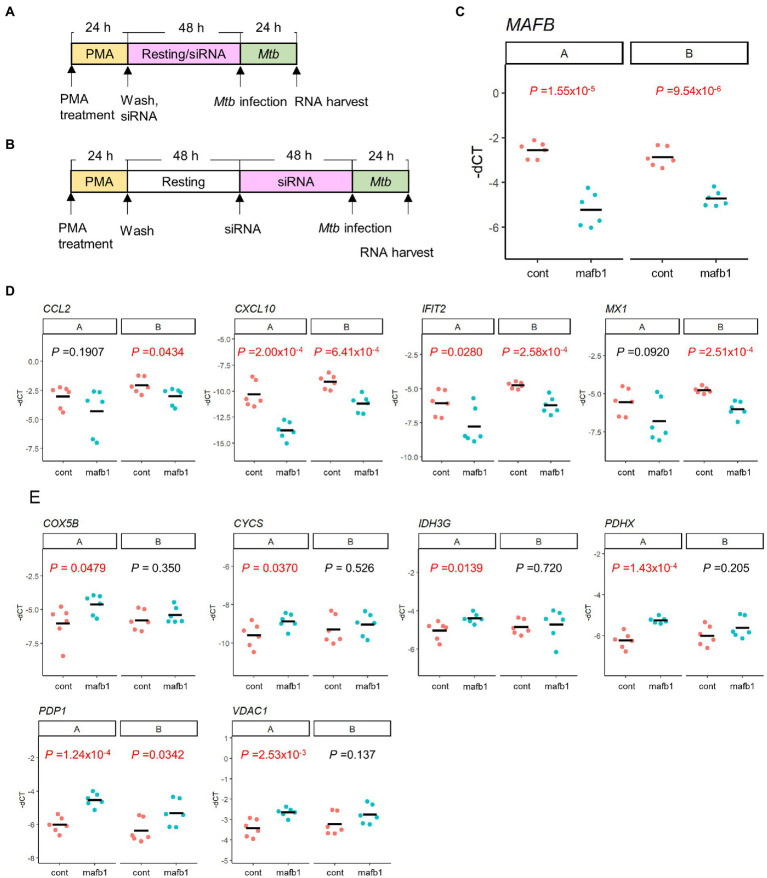
The effect of siRNA treatment on gene expression related to IFN responses and metabolism. **(A,B)** Transfection of siRNA was carried out during the resting period following PMA stimulation **(A)** or after the completion of the resting period **(B)**. **(C–E)** mRNA levels of *MAFB* **(C)**, IFN-γ related genes **(D)**, and metabolism related genes **(E)** in MAFB-KD (mafb1) or control (cont) macrophages with *Mtb* infection under both conditions were examined by qRT–PCR. The-dCT values of the indicated gene are shown. *p* values calculated using Student’s *t* test are also shown.

In this study, we conducted MAFB gene depletion during the resting period following PMA stimulation (Figure 9A). Because MAFB is involved in macrophage differentiation (Sieweke et al., 1996; Sarrazin et al., 2009), it is possible that the variability of gene expression profiles by MAFB depletion is caused by inhibition of macrophage differentiation. To rule this possibility out, we performed siRNA transfection after resting cells (Figure 9B) and compared the gene expression related to IFN responses and metabolism between the two conditions. We confirmed successful MAFB knockdown under both conditions (Figure 9C). We found that the expression profiles of these genes under both conditions exhibited similar trends; MAFB depletion depressed the expression of genes related to IFN responses and enhanced the expression of genes related to metabolism in *Mtb*-infected macrophages (Figures 9D,E). These results suggest that MAFB regulates several important pathways in response to *Mtb* infection.

## Discussion

MAFB plays a key role in regulating macrophage differentiation (Sieweke et al., 1996; Sarrazin et al., 2009) and has been implicated in various macrophage-related diseases (Hamada et al., 2020). In *Mtb* infection, one report of a GWAS suggested the association of *MAFB* with TB onset (Mahasirimongkol et al., 2012); however, the detailed function of MAFB in TB immunity has not been elucidated. First, we investigated the level of MAFB expression in different states of macrophages. As previous reports demonstrated in mice (Aziz et al., 2006; Lavin et al., 2014), MAFB was highly and specifically expressed in macrophages (Figure 1). In the present study, we compared genome-wide transcriptional profiles between MAFB-KD and control macrophages upon *Mtb* infection (Figure 5). Generally, IFN-γ secreted by natural killer cells and T cells activates macrophages and promotes bactericidal activities (Korbel et al., 2008). Therefore, we also compared upon *Mtb* infection when macrophages were activated by IFN-γ (Supplementary Figure S1). To investigate the effect of MAFB knockdown on IFN-γ stimulation, we focused on the activity of IFN-γ-inducible genes (Figure 8).

The role of cytokines, especially IFN-γ, in host defense against *Mtb* infection has been well studied (Zhang et al., 2008). Cooper et al. demonstrated that IFN-γ-deficient mice show high susceptibility to *Mtb* infection (Cooper et al., 1993). The association of type I IFNs in *Mtb* infection has been discussed as well as IFN-γ. Type I IFNs are thought to have beneficial effects during viral infection, but this is not always the case in *Mtb* infection (Moreira-Teixeira et al., 2018). Some studies have suggested dual pro- and anti-inflammatory properties of type I IFNs during *Mtb* infection in a time-dependent manner or in relation to type II IFNs (Adler and Adler, 2021). Desvignes et al. showed that mice lacking both the type I and type II IFN receptor genes, *Ifnar* and *Ifngr*, respectively, developed widespread granulomatous lesions, leading to early death, indicating a detrimental outcome of *Mtb* infection caused by disruption of both type I and type II IFNs (Desvignes et al., 2012). Herein, we found that the activity of both type I and type II IFNs was suppressed by MAFB knockdown during *Mtb* infection. *Mtb* infection and IFN-γ activation synergistically induced genes that are responsible for both type I and type II IFN responses but the induction was impaired by MAFB knockdown (Figure 6A), suggesting that MAFB mediates the IFN signaling pathway.

Regarding the involvement of MAFB in type I IFN expression, Kim and Seed showed that MAFB exhibits both stimulatory and inhibitory activities on type I IFN induction (Kim and Seed, 2010). Saiga et al. demonstrated MafB as a negative regulator of the type I IFN response in plasmacytoid dendritic cells (Saiga et al., 2022). However, Vega et al. demonstrated the repressed expression of type I IFN response-related genes in MAFB-KD human monocyte-derived macrophages (Vega et al., 2020), which is consistent with our result where similar repression was observed in MAFB-KD THP-1-derived macrophages infected with *Mtb*. Combined with the evidence that MAFB is specifically expressed in macrophages within the hematopoietic lineage (Lavin et al., 2014), the different cell types and types of stimulation used in previous studies explain the human macrophage specific role of MAFB in the type I IFN response in our data.

Chemokines play major important roles in limiting bacterial spread in *Mtb*-infected mice and humans; e.g., CCL2 and CCL7 are essential for recruiting macrophages and T lymphocytes to form granulomas in the lungs (Chensue et al., 1996; Warmington et al., 1999; Shang et al., 2000; Qiu et al., 2001). Due to a delay or a lack of immune cell recruitment, CCR2-deficient mice developed loosely formed granulomas; however, the bacterial burden was not greatly impacted (Scott and Flynn, 2002). CXCL10 and CXCL11 bind to CXCR3, which is primarily expressed by activated CD4^+^ and CD8^+^ T cells (Tannenbaum et al., 1998). Effector T cells expressing CXCR3 are found within granulomas and are associated with granuloma formation through neutrophil recruitment *via* CXCR3 signaling (Seiler et al., 2003). Not only the gene expression of these chemokines but also secreted chemokine levels were decreased in *Mtb-*infected MAFB-KD macrophages (Figure 8). These studies show that impaired chemokine activity makes a host vulnerable to *Mtb* infection. Thus, we expected that MAFB-KD macrophages would show a higher bacterial burden; however, no significant differences in CFU were observed in our experimental model (Figure 3). Considering the impairment of phagocytic activity and the same numbers of viable bacilli, MAFB-KD macrophages represent a more permissive niche for bacterial growth. Furthermore, decreased chemokine activities and phagocytosis of *Mtb* in macrophages is thought to reduce the recruitment of monocytes and macrophages to the infected loci and the containment activity of infected *Mtb*. Our findings suggest that knockdown of MAFB has an unfavorable effect on *Mtb* control in macrophages.

In addition to the effect of MAFB knockdown on the immune response, we found that genes related to oxidative phosphorylation were upregulated in *Mtb*-infected MAFB-KD macrophages (Figure 6B). In *Mtb*-infected alveolar macrophages, oxidative phosphorylation, mitochondrial functions and lipid uptake are enhanced, supporting *Mtb* growth with lipid-rich nutrients (Pisu et al., 2020). Foamy macrophages are the key feature of TB granulomas and are also the niche for *Mtb,* which utilizes cytosolic lipids for energy synthesis (Shim et al., 2020). Thus, controlling metabolic changes in macrophages is a potential strategy for host-directed therapy. MAFB has been reported to promote atherosclerosis by inhibiting apoptosis of foam-cells in a mouse model (Hamada et al., 2014). The upregulation of oxidative phosphorylation and adipogenesis gene sets showed the engagement of MAFB in macrophage metabolism, suggesting that MAFB is a potential host-directed therapeutic target for TB.

In conclusion, the data presented here demonstrated the involvement of MAFB in the immune responses of human macrophages to *Mtb* infection, including type I and II IFN activity, and the activity of inflammatory chemokines, which are important in controlling TB. Our findings suggest that understanding MAFB and its related pathways can provide a breakthrough in understanding the complexity of host TB immunity and in the search for potential targets for the diagnosis or host-directed therapy of TB.

## Data availability statement

The datasets presented in this study can be found in online repositories. The names of the repository/repositories and accession number(s) can be found at: https://ddbj.nig.ac.jp/resource/sra-submission/DRA014120.

## Author contributions

HH, SS, MH, and NK designed the project and analyzed the data, and wrote and revised the manuscript. HH, SS, KW, and MH performed the experiments. All authors contributed to the article and approved the submitted version.

## Funding

This study was supported by the Science and Technology Research Partnership for Sustainable Development (18jm0110010h0205), JSPS KAKENHI (JP20K16259) and the Emerging/Re-emerging Infectious Diseases Project of the Japan Agency for Medical Research and Development (21fk0108090, and 22wm0225011).

## Conflict of interest

The authors declare that the research was conducted in the absence of any commercial or financial relationships that could be construed as a potential conflict of interest.

## Publisher’s note

All claims expressed in this article are solely those of the authors and do not necessarily represent those of their affiliated organizations, or those of the publisher, the editors and the reviewers. Any product that may be evaluated in this article, or claim that may be made by its manufacturer, is not guaranteed or endorsed by the publisher.

## Footnotes

^1^ https://github.com/FelixKrueger/TrimGalore

^2^ https://github.com/broadinstitute/picard

^3^ https://cran.r-project.org/web/packages/pheatmap/index.html

## Supplementary material

SUPPLEMENTARY FIGURE S1 Transcriptomic analysis of MAFB-KD macrophages infected with Mtb in the presence of IFN-γ. **(A)** mRNA-seq was performed to show the impact of MAFB knockdown under conditions of Mtb infection with IFN-γ activation. PMA-stimulated THP-1 cells transfected with mafb or control siRNA and then activated with IFN-γ were infected with Mtb for 24 h. MA plot showing the DEGs in Mtb-infected IFN-γ-activated MAFB-KD macrophages compared with control macrophages marked in red (FDR < 0.01). **(B)** GOBP enrichment analyses of upregulated (DEG_up) and downregulated DEGs (DEG_down) in Mtb-infected IFN-γ-activated MAFB-KD macrophages. The 10 most significant GOBP terms are shown. **(C)** GSEA of Mtb-infected IFN-γ-activated MAFB-KD cells. Enriched hallmarks are shown (FDR < 0.25). The color scale indicates the adjusted *P* value, and the bar size in the histogram size indicates the NES. Count, gene count. FC, fold change. CPM, counts per million. NES, normalized enrichment score. padj, adjusted *P* value.

SUPPLEMENTARY FIGURE S2 Comparison of MAFB targeted genes obtained from mRNA-seq in our study and ChIP-seq. **(A)** Venn diagram comparing the numbers of genes for DEGs in MAFB-KD THP-1 macrophages infected with Mtb (Figure 5) and genes bound by MafB in mouse macrophages (Dieterich et al., 2015). Of 6768 DEGs, 2726 genes (40.3%) were considered as direct targets of MAFB. **(B)** GOBP enrichment analysis of direct target genes for MAFB in DEGs. The 10 most significant GOBP terms are shown (*P* value < 0.1). Gene Ratio: gene ratio, Count: gene count, padjust; adjusted *P* value.

## References

[ref1] Abel L. Fellay J. Haas D. W. Schurr E. Srikrishna G. Urbanowski M.. (2018). Genetics of human susceptibility to active and latent tuberculosis: present knowledge and future perspectives. Lancet Infect. Dis. 18, e64–e75. doi: 10.1016/S1473-3099(17)30623-0, PMID: 29111156 PMC8903186

[ref2] Adler B. Adler H. (2021). Type I interferon signaling and macrophages: a double-edged sword? Cell. Mol. Immunol. 19, 967–968. doi: 10.1038/s41423-020-00609-0, PMID: 33420351 PMC9424296

[ref3] Ashburner M. Ball C. A. Blake J. A. Botstein D. Butler H. Cherry J. M.. (2000). Gene ontology: tool for the unification of biology. The Gene Ontology Consortium. Nat Genet 25, 25–29. doi: 10.1038/75556, PMID: 10802651 PMC3037419

[ref4] Aziz A. Vanhille L. Mohideen P. Kelly L. M. Otto C. Bakri Y.. (2006). Development of macrophages with altered actin organization in the absence of Maf B. Mol. Cell. Biol. 26, 6808–6818. doi: 10.1128/MCB.00245-06, PMID: 16943423 PMC1592864

[ref5] Chanput W. Mes J. J. Wichers H. J. (2014). THP-1 cell line: an in vitro cell model for immune modulation approach. Int. Immunopharmacol. 23, 37–45. doi: 10.1016/j.intimp.2014.08.002, PMID: 25130606

[ref6] Chen Y. Lun A. T. Smyth G. K. (2016). From reads to genes to pathways: differential expression analysis of RNA-Seq experiments using Rsubread and the edgeR quasi-likelihood pipeline. F1000Res 5:1438. doi: 10.12688/f1000research.8987.2 27508061 PMC4934518

[ref7] Chensue S. W. Warmington K. S. Ruth J. H. Sanghi P. S. Lincoln P. Kunkel S. L. (1996). Role of monocyte chemoattractant protein-1 (MCP-1) in Th1 (mycobacterial) and Th2 (schistosomal) antigen-induced granuloma formation: relationship to local inflammation, Th cell expression, and IL-12 production. J. Immunol. 157, 4602–4608. 8906839

[ref8] Cooper A. M. Dalton D. K. Stewart T. A. Griffin J. P. Russell D. G. Orme I. M. (1993). Disseminated tuberculosis in interferon gamma gene-disrupted mice. J. Exp. Med. 178, 2243–2247. doi: 10.1084/jem.178.6.2243, PMID: 8245795 PMC2191280

[ref9] Dallmann-Sauer M. Correa-Macedo W. Schurr E. (2018). Human genetics of mycobacterial disease. Mamm. Genome 29, 523–538. doi: 10.1007/s00335-018-9765-4, PMID: 30116885 PMC6132723

[ref10] Desvignes L. Wolf A. J. Ernst J. D. (2012). Dynamic roles of type I and type II IFNs in early infection with mycobacterium tuberculosis. J. Immunol. 188, 6205–6215. doi: 10.4049/jimmunol.1200255, PMID: 22566567 PMC3370955

[ref11] Dheda K. Perumal T. Moultrie H. Perumal R. Esmail A. Scott A. J.. (2022). The intersecting pandemics of tuberculosis and COVID-19: population-level and patient-level impact, clinical presentation, and corrective interventions. Lancet Respir. Med. 10, 603–622. doi: 10.1016/S2213-2600(22)00092-3 35338841 PMC8942481

[ref12] Dieterich L. C. Klein S. Mathelier A. Sliwa-Primorac A. Ma Q. Hong Y. K.. (2015). DeepCAGE Transcriptomics reveal an important role of the transcription factor MAFB in the lymphatic endothelium. Cell Rep. 13, 1493–1504. doi: 10.1016/j.celrep.2015.10.002, PMID: 26549461

[ref13] Furuuchi K. Seto S. Nakamura H. Hikichi H. Miyabayashi A. Wakabayashi K.. (2022). Novel screening system of virulent strains for the establishment of a Mycobacterium avium complex lung disease mouse model using whole-genome sequencing. Microbiol Spectr 10:e0045122. doi: 10.1128/spectrum.00451-22, PMID: 35579455 PMC9241706

[ref14] Hamada M. Nakamura M. Tran M. T. Moriguchi T. Hong C. Ohsumi T.. (2014). MafB promotes atherosclerosis by inhibiting foam-cell apoptosis. Nat. Commun. 5:3147. doi: 10.1038/ncomms4147, PMID: 24445679

[ref15] Hamada M. Tsunakawa Y. Jeon H. Yadav M. K. Takahashi S. (2020). Role of MafB in macrophages. Exp. Anim. 69, 1–10. doi: 10.1538/expanim.19-0076, PMID: 31582643 PMC7004803

[ref16] Kataoka K. Fujiwara K. T. Noda M. Nishizawa M. (1994). MafB, a new Maf family transcription activator that can associate with Maf and Fos but not with Jun. Mol. Cell. Biol. 14, 7581–7591. PMID: 7935473 10.1128/mcb.14.11.7581-7591.1994 PMC359294

[ref17] Kelly L. M. Englmeier U. Lafon I. Sieweke M. H. Graf T. (2000). MafB is an inducer of monocytic differentiation. EMBO J. 19, 1987–1997. doi: 10.1093/emboj/19.9.1987, PMID: 10790365 PMC305687

[ref18] Kim D. Paggi J. M. Park C. Bennett C. Salzberg S. L. (2019). Graph-based genome alignment and genotyping with HISAT2 and HISAT-genotype. Nat. Biotechnol. 37, 907–915. doi: 10.1038/s41587-019-0201-4, PMID: 31375807 PMC7605509

[ref19] Kim H. Seed B. (2010). The transcription factor MafB antagonizes antiviral responses by blocking recruitment of coactivators to the transcription factor IRF3. Nat. Immunol. 11, 743–750. doi: 10.1038/ni.1897, PMID: 20581830 PMC3050627

[ref20] Korbel D. S. Schneider B. E. Schaible U. E. (2008). Innate immunity in tuberculosis: myths and truth. Microbes Infect. 10, 995–1004. doi: 10.1016/j.micinf.2008.07.039, PMID: 18762264

[ref21] Korotkevich G. Sukhov V. Budin N. Shpak B. Artyomov M. N. Sergushichev A. (2021). Fast gene set enrichment analysis. bioRxiv:060012. doi: 10.1101/060012

[ref22] Lavin Y. Winter D. Blecher-Gonen R. David E. Keren-Shaul H. Merad M.. (2014). Tissue-resident macrophage enhancer landscapes are shaped by the local microenvironment. Cells 159, 1312–1326. doi: 10.1016/j.cell.2014.11.018, PMID: 25480296 PMC4437213

[ref23] Liao Y. Smyth G. K. Shi W. (2014). featureCounts: an efficient general purpose program for assigning sequence reads to genomic features. Bioinformatics 30, 923–930. doi: 10.1093/bioinformatics/btt656, PMID: 24227677

[ref24] Liu T. M. Wang H. Zhang D. N. Zhu G. Z. (2019). Transcription factor MafB suppresses type I interferon production by CD14(+) monocytes in patients with chronic hepatitis C. Front. Microbiol. 10:1814. doi: 10.3389/fmicb.2019.01814, PMID: 31447817 PMC6692491

[ref25] Mahasirimongkol S. Yanai H. Mushiroda T. Promphittayarat W. Wattanapokayakit S. Phromjai J.. (2012). Genome-wide association studies of tuberculosis in Asians identify distinct at-risk locus for young tuberculosis. J. Hum. Genet. 57, 363–367. doi: 10.1038/jhg.2012.35, PMID: 22551897

[ref26] Moni M. A. Lio P. (2017). Genetic profiling and comorbidities of Zika infection. J. Infect. Dis. 216, 703–712. doi: 10.1093/infdis/jix327, PMID: 28934431

[ref27] Moreira-Teixeira L. Mayer-Barber K. Sher A. O'garra A. (2018). Type I interferons in tuberculosis: foe and occasionally friend. J. Exp. Med. 215, 1273–1285. doi: 10.1084/jem.20180325, PMID: 29666166 PMC5940272

[ref28] Orlova M. Cobat A. Huong N. T. Ba N. N. Van Thuc N. Spencer J.. (2013). Gene set signature of reversal reaction type I in leprosy patients. PLoS Genet. 9:e1003624. doi: 10.1371/journal.pgen.1003624, PMID: 23874223 PMC3708838

[ref29] Pisu D. Huang L. Grenier J. K. Russell D. G. (2020). Dual RNA-Seq of Mtb-infected macrophages in vivo reveals ontologically distinct host-pathogen interactions. Cell Rep. 30:e334, 335–350. doi: 10.1016/j.celrep.2019.12.033 PMC7032562 31940480

[ref30] Pu W. Zhao C. Wazir J. Su Z. Niu M. Song S.. (2021). Comparative transcriptomic analysis of THP-1-derived macrophages infected with mycobacterium tuberculosis H37Rv, H37Ra and BCG. J. Cell. Mol. Med. 25, 10504–10520. doi: 10.1111/jcmm.16980, PMID: 34632719 PMC8581329

[ref31] Qiu B. Frait K. A. Reich F. Komuniecki E. Chensue S. W. (2001). Chemokine expression dynamics in mycobacterial (type-1) and schistosomal (type-2) antigen-elicited pulmonary granuloma formation. Am. J. Pathol. 158, 1503–1515. doi: 10.1016/S0002-9440(10)64101-6, PMID: 11290568 PMC1891908

[ref32] Robinson M. D. Mccarthy D. J. Smyth G. K. (2010). edgeR: a bioconductor package for differential expression analysis of digital gene expression data. Bioinformatics 26, 139–140. doi: 10.1093/bioinformatics/btp616, PMID: 19910308 PMC2796818

[ref33] Saiga H. Ueno M. Tanaka T. Kaisho T. Hoshino K. (2022). Transcription factor MafB-mediated inhibition of type I interferons in plasmacytoid dendritic cells. Int. Immunol. 34, 159–172. doi: 10.1093/intimm/dxab103, PMID: 34734243

[ref34] Sarrazin S. Mossadegh-Keller N. Fukao T. Aziz A. Mourcin F. Vanhille L.. (2009). MafB restricts M-CSF-dependent myeloid commitment divisions of hematopoietic stem cells. Cells 138, 300–313. doi: 10.1016/j.cell.2009.04.057, PMID: 19632180

[ref35] Satproedprai N. Wichukchinda N. Suphankong S. Inunchot W. Kuntima T. Kumpeerasart S.. (2015). Diagnostic value of blood gene expression signatures in active tuberculosis in Thais: a pilot study. Genes Immun. 16, 253–260. doi: 10.1038/gene.2015.4, PMID: 25764116

[ref36] Scott H. M. Flynn J. L. (2002). Mycobacterium tuberculosis in chemokine receptor 2-deficient mice: influence of dose on disease progression. Infect. Immun. 70, 5946–5954. doi: 10.1128/IAI.70.11.5946-5954.2002, PMID: 12379669 PMC130313

[ref37] Seiler P. Aichele P. Bandermann S. Hauser A. E. Lu B. Gerard N. P.. (2003). Early granuloma formation after aerosol mycobacterium tuberculosis infection is regulated by neutrophils via CXCR3-signaling chemokines. Eur. J. Immunol. 33, 2676–2686. doi: 10.1002/eji.200323956, PMID: 14515251

[ref38] Seto S. Nakamura H., Guo, TC., Hikichi H. Wakabayashi K. Miyabayashi A. Nagata T. Hijikata M. Keicho N. (2022). Spatial multiomic profiling reveals the novel polarization of foamy macrophages within necrotic granulomatous lesions developed in lungs of C3HeB/FeJ mice infected with mycobacterium tuberculosis. Front. Cell. Infect. Microbiol. 12:968543, doi: 10.3389/fcimb.2022.968543 36237431 PMC9551193

[ref39] Seto S. Tsujimura K. Koide Y. (2012). Coronin-1a inhibits autophagosome formation around mycobacterium tuberculosis-containing phagosomes and assists mycobacterial survival in macrophages. Cell. Microbiol. 14, 710–727. doi: 10.1111/j.1462-5822.2012.01754.x, PMID: 22256790

[ref40] Shang X. Qiu B. Frait K. A. Hu J. S. Sonstein J. Curtis J. L.. (2000). Chemokine receptor 1 knockout abrogates natural killer cell recruitment and impairs type-1 cytokines in lymphoid tissue during pulmonary granuloma formation. Am. J. Pathol. 157, 2055–2063. 11106578 10.1016/S0002-9440(10)64844-4 PMC1885763

[ref41] Shim D. Kim H. Shin S. J. (2020). Mycobacterium tuberculosis infection-driven foamy macrophages and their implications in tuberculosis control as targets for host-directed therapy. Front. Immunol. 11:910. doi: 10.3389/fimmu.2020.00910, PMID: 32477367 PMC7235167

[ref42] Sieweke M. H. Tekotte H. Frampton J. Graf T. (1996). MafB is an interaction partner and repressor of Ets-1 that inhibits erythroid differentiation. Cells 85, 49–60. doi: 10.1016/S0092-8674(00)81081-8, PMID: 8620536

[ref43] Subramanian A. Tamayo P. Mootha V. K. Mukherjee S. Ebert B. L. Gillette M. A.. (2005). Gene set enrichment analysis: a knowledge-based approach for interpreting genome-wide expression profiles. Proc. Natl. Acad. Sci. U. S. A. 102, 15545–15550. doi: 10.1073/pnas.0506580102, PMID: 16199517 PMC1239896

[ref44] Tannenbaum C. S. Tubbs R. Armstrong D. Finke J. H. Bukowski R. M. Hamilton T. A. (1998). The CXC chemokines IP-10 and Mig are necessary for IL-12-mediated regression of the mouse RENCA tumor. J. Immunol. 161, 927–932. 9670971

[ref45] Vega M. A. Simon-Fuentes M. Gonzalez De La Aleja A. Nieto C. Colmenares M. Herrero C.. (2020). MAFB and MAF transcription factors as macrophage checkpoints for COVID-19 severity. Front. Immunol. 11:603507. doi: 10.3389/fimmu.2020.603507, PMID: 33312178 PMC7708330

[ref46] Warmington K. S. Boring L. Ruth J. H. Sonstein J. Hogaboam C. M. Curtis J. L.. (1999). Effect of C-C chemokine receptor 2 (CCR2) knockout on type-2 (schistosomal antigen-elicited) pulmonary granuloma formation: analysis of cellular recruitment and cytokine responses. Am. J. Pathol. 154, 1407–1416. doi: 10.1016/S0002-9440(10)65394-1, PMID: 10329593 PMC1866581

[ref47] WHO. (2021). Global tuberculosis report 2021. (Geneva, World Health Organization).

[ref48] Wu K. Dong D. Fang H. Levillain F. Jin W. Mei J.. (2012). An interferon-related signature in the transcriptional core response of human macrophages to mycobacterium tuberculosis infection. PLoS One 7:e38367. doi: 10.1371/journal.pone.0038367, PMID: 22675550 PMC3366933

[ref49] Yamada H. Mizuno S. Reza-Gholizadeh M. Sugawara I. (2001). Relative importance of NF-kappaB p50 in mycobacterial infection. Infect. Immun. 69, 7100–7105. doi: 10.1128/IAI.69.11.7100-7105.2001, PMID: 11598086 PMC100095

[ref50] Yu G. Wang L. G. Han Y. He Q. Y. (2012). Cluster profiler: an R package for comparing biological themes among gene clusters. OMICS 16, 284–287. doi: 10.1089/omi.2011.0118, PMID: 22455463 PMC3339379

[ref51] Zhang S. Y. Boisson-Dupuis S. Chapgier A. Yang K. Bustamante J. Puel A.. (2008). Inborn errors of interferon (IFN)-mediated immunity in humans: insights into the respective roles of IFN-alpha/beta, IFN-gamma, and IFN-lambda in host defense. Immunol. Rev. 226, 29–40. doi: 10.1111/j.1600-065X.2008.00698.x, PMID: 19161414

