# Supplementary figures and images for: Transcription factor MAFB controls type I and II interferon response-mediated host immunity in Mycobacterium tuberculosis-infected macrophages
Source: Front Microbiol. 2022 Nov 3;13:962306. doi: 10.3389/fmicb.2022.962306 (PMC9670303; doi:10.3389/fmicb.2022.962306)

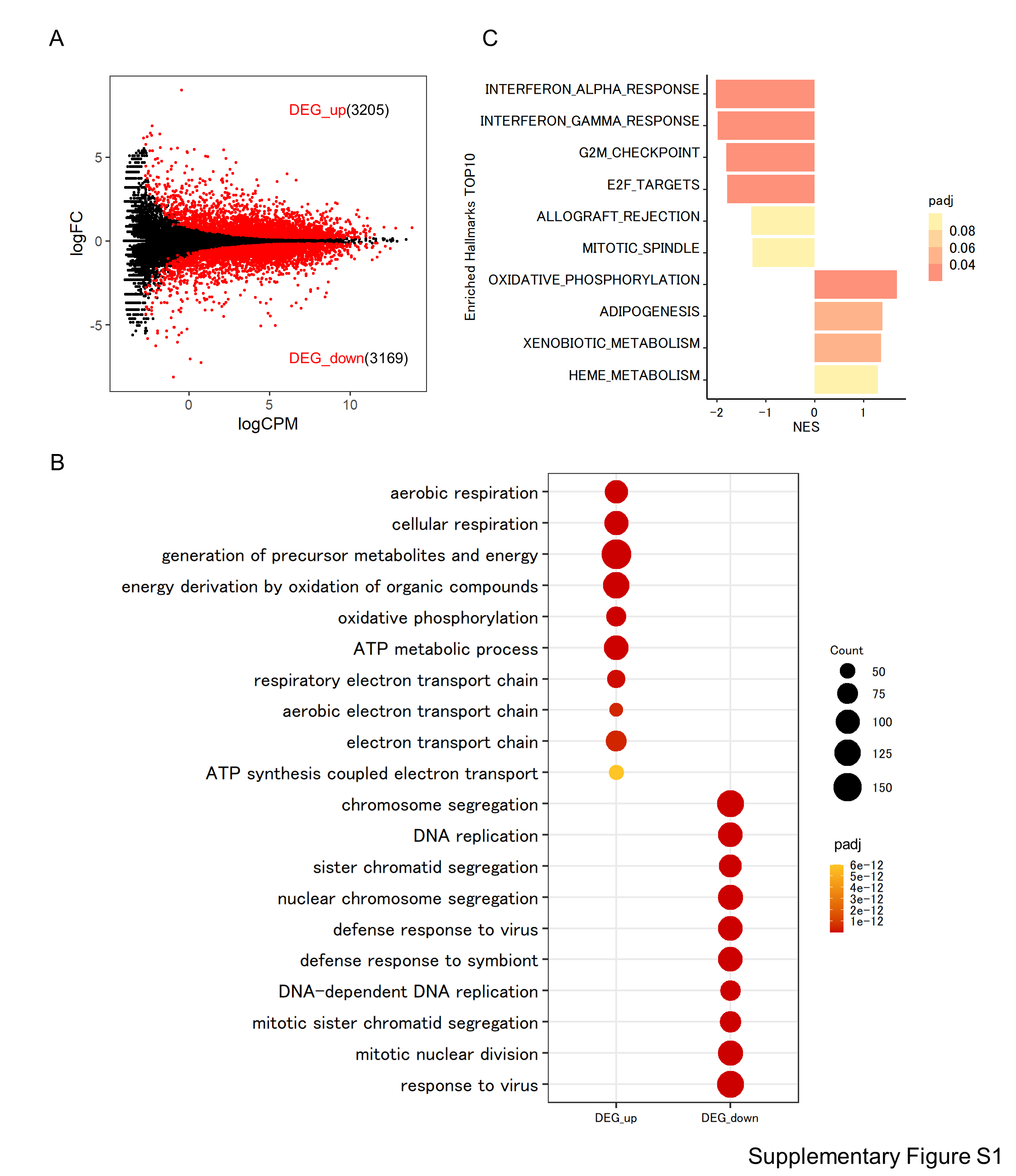

Supplement: SUPPLEMENTARY FIGURE S1 — Transcriptomic analysis of MAFB-KD macrophages infected with Mtb in the presence of IFN-γ. (A) mRNA-seq was performed to show the impact of MAFB knockdown under conditions of Mtb infection with IFN-γ activation. PMA-stimulated THP-1 cells transfected with mafb or control siRNA and then activated with IFN-γ were infected with Mtb for 24 h. MA plot showing the DEGs in Mtb-infected IFN-γ-activated MAFB-KD macrophages compared with control macrophages marked in red (FDR < 0.01). (B) GOBP enrichment analyses of upregulated (DEG_up) and downregulated DEGs (DEG_down) in Mtb-infected IFN-γ-activated MAFB-KD macrophages. The 10 most significant GOBP terms are shown. (C) GSEA of Mtb-infected IFN-γ-activated MAFB-KD cells. Enriched hallmarks are shown (FDR < 0.25). The color scale indicates the adjusted P value, and the bar size in the histogram size indicates the NES. Count, gene count. FC, fold change. CPM, counts per million. NES, normalized enrichment score. padj, adjusted P value. [file Image_1.TIF]

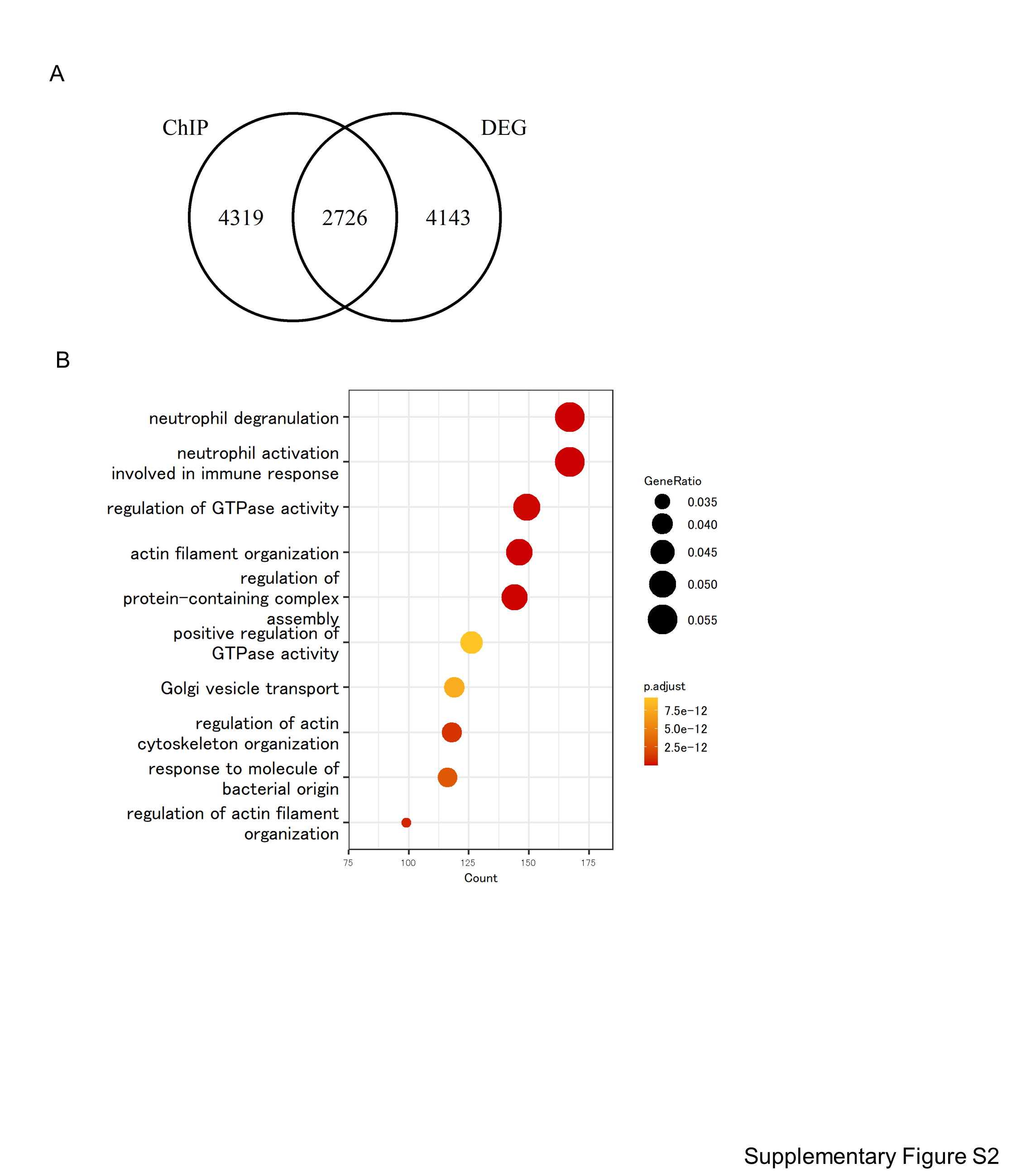

Supplement: SUPPLEMENTARY FIGURE S2 — Comparison of MAFB targeted genes obtained from mRNA-seq in our study and ChIP-seq. (A) Venn diagram comparing the numbers of genes for DEGs in MAFB-KD THP-1 macrophages infected with Mtb (Figure 5) and genes bound by MafB in mouse macrophages (Dieterich et al., 2015). Of 6768 DEGs, 2726 genes (40.3%) were considered as direct targets of MAFB. (B) GOBP enrichment analysis of direct target genes for MAFB in DEGs. The 10 most significant GOBP terms are shown (P value < 0.1). Gene Ratio: gene ratio, Count: gene count, padjust; adjusted P value. [file Image_2.TIF]
