# Supplementary material for: Transcription factor MAFB controls type I and II interferon response-mediated host immunity in Mycobacterium tuberculosis-infected macrophages
Source: Front Microbiol. 2022 Nov 3;13:962306. doi: 10.3389/fmicb.2022.962306 (PMC9670303; doi:10.3389/fmicb.2022.962306)
Supplement: Supplementary file 3 [file Table_1.PDF]

Supplementary Material

Target gene	Forward Primer (5´ to 3´)	Reverse Primer (5´ to 3´)	Probe (5´ to 3´)
C10orf10	GGCTTCCTTTGGACTGGA	CCTCATCACTCTGGCGAGA	TCCTCAGCTCCAGGTTGCCAC
CCL2	AGCAGCCACCTTCATTCC	GCCTCTGCATGAGATCTTC	TCAATGCCCCAGTCACCTGCT
CCL7	AGCCTTGGATGTATATGTCATCTC	ACAATACCCCATGAGGTAGA	AGGAGCATCCCACAGTTTTTACAGCA
CCL8	GGTCCTCCCATGGATCATCA	CAACAGCAGTCAAAGCACAC	ACTGATTGCCAAAGAATACCAAAGTGTTTCAC
CD163	GCATAGTAACTGTACTCACCAACA	CCAGAACACATATTCCCTCCAC	ATCCATCTGAGCAGGTCACTCCAG
CHODL	AATGAAGTCAGCTGGAGTTGG	CTGGACAGTTCATGGAAGTAGG	CTGTGTGCCGAACCTGCAGTC
CPA4	CGAAATCCTGGAAGCTCCTG	GATTTCACCTCCACTTCCGA	CTGGCTCCCTTTCCTGCAAAACT
CXCL10	GACATATTCTGAGCCTACAGCA	CAGTTCTAGAGAGAGGTACTCCT	ACCTCCAGTCTCAGCACCATGAATC
CXCL11	TATTGTGTGCTACAGTTGTTCAAG	GGTACATTATGGAGGCTTTCTCA	TGCCACTTTCACTGCTTTTACCCCA
CXCL12	TGCATTGACCCGAAGCTAA	CCCTTCCCTAACACTGGTTTC	CGTCTGACCCTCTCACATCTTGAACC
GAPDH	ACATCGCTCAGACACCATG	TGTAGTTGAGGTCAATGAAGGG	AAGGTCGGAGTCAACGGATTTGGTC
IGFN	ACAGGAAGGACTACGAGAAGA	GCTGGAGATGGTGTTGATGT	AGGAGATGAAGAAGGAACAGGAGGACA
IL36B	TCCCCACTCTGTCTTTCTCA	TGTCTTCAGAGCCTTTTGTGA	CTTTTCCTAGCCTCCTCACCACCA
IL36RN	AGGAGAAAGGAACATTCTGAGG	TATGCAGATAAAGCACCTTCAATG	CTTCATTCGGAAGCAGCGCC
MAFB	CTTTCTGAACTTTGCGCGTTA	CCGTCTGTCCTTCCTTCTTTC	TCCCTCTCCTTTCCTCGTTGCTCT
MMP8	GCTTCCATTTCTGCTCTTACTC	GCCATTCTTCCTTGTAGACTGA	CTGGTTGCTTGGTAATTGGTAGAACTTTTCC

Target gene	Forward Primer (5´ to 3´)	Reverse Primer (5´ to 3´)	Probe (5´ to 3´)
COX5B	CAATGGCTTCAAGGTTACTTCG	CGCCTGCTCTTCATCAGTG	CCATGGCATCTGGAGGTGGTGT
CYCS	AATCCGTCCACTTGCCTTG	GGGCTGATTACTTCTGGGTTA	CTCACGCCTGTAATCCCAGCACTT
IDH3G	CGTGAGTTCCAATGCTGATG	GCTGGTGCGAAGGATGT	TTCGAGATTTGTGCGACGGTGGC
PDHX	CTAATGCCATCACTGTCTCCTAC	AGCTTTGTCAGTCTCAATTTCAC	CGCTTCACCTTCCTTTTTCAGCCAT
PDP1	GGAAGAATCGTTTGGTCTCCT	CCATAGATCCTGCTCAGTTCAC	TGCCACTGTTCTCTGATGCCATGC
VDAC1	CTTCGATTCATCCTTCTCACCT	AGCAATGTCGAAATCCATGTC	CTCCCGCTTGTACCCTGTCTTGATT

Supplementary Table S1. Target genes and the primers used for qRT–PCR.
